# The opening of phenome-assisted selection era in the early seedling stage

**DOI:** 10.1038/s41598-019-46405-3

**Published:** 2019-07-09

**Authors:** Song Lim Kim, Yong Suk Chung, Renato Rodrigues Silva, Hyeonso Ji, Hongseok Lee, Inchan Choi, Nyunhee Kim, Eungyeong Lee, JeongHo Baek, Gang-Seob Lee, Taek-Ryoun Kwon, Kyung-Hwan Kim

**Affiliations:** 1 National Institute of Agricultural Sciences, Rural Development Administration (RDA), Jeonju, 54874 Republic of Korea; 2 Department of Plant Resources and Environment, Jeju National University, 63243, Jeju, Republic of Korea; 3 Institute of Mathematics and Statistics, Federal University of Goiás, Goiânia, Brazil

**Keywords:** Plant breeding, Plant breeding, Plant genetics, Plant genetics

## Abstract

Faster and more efficient breeding cycle is not an option to deal with unpredictable and fast global climate changes. Phenomics for collecting huge number of individuals in accurate manner could be an answer to solve this problem. We collected image data to measure plant height and manual data for shoot length to be compared. QTLs clustered of plant height and shoot length were detected in 2-week old seedlings, which was consistent with many other reports using various genetic resources in matured stage. Further, these traits are highly correlated with yield by pleiotropism or tight linkage of those traits. It implies the “phenome-assisted selection” can be applied for yield trait in rice in the very early stage to shorten the breeding cycle significantly in fast but low-cost manner.

## Introduction

Recent radical climate changes demand faster and more efficient breeding cycle. Fortunately, newly emerging and fast developing area, phenomics, take attentions in this circumstance. It enables the target phenotypic traits to be collected in the accurate and repeatable manner in large scale. However, this technology cannot reduce the life cycle time of any crops. To shorten the breeding cycle even further, developing new method is required to screen traits, which associated with the target traits such as yield, in the early growth stage. This is true for any breeding target. The initial stage of breeding requires very large number of populations. In general, the final goal of breeding is high yield. Thus, screening the high yield potential individuals before screening may save a lot of time, resources, and effort; this can be very true especially in the case of rice because large number of non-vigor seedling could be eliminated before transplanting.

Seedling vigor in rice (*Oryza sativa* L.) is associated with many traits such as plant viability, height, thickness of stems, and uniformity^1^. Further, it is known indicator for increasing quality of tillering and yield^2^. It could be expressed by several components such as primary/secondary tiller, shoot length, biomass, and leaf area index. The efficient screening method for seedling vigor using any suitable evaluating components could be useful for selecting elite lines for high yield potential. Those components in the early stage of rice growth has to be collected in a short period before the next stage comes. However, the conventional screening methods cannot achieve this in the short period. More importantly, they cannot screen the large-scale population, which is essential to accelerate breeding cycle. To overcome the problems in the conventional screening methods, the current study evaluated three components for seedling vigor including projected plant height using image analysis as well as shoot length and fresh weight manually to compare with image data. QTL analysis was followed to reveal the loci that are associated with those collected traits. The reason why plant height was targeted for high throughput phenotyping was because it is relatively easy to measure and correlated with yield of rice^3^. Here we are excited to report the opening of phenome-assisted selection for initial screening for high yield lines in the breeding program and accelerate the speed of breeding.

We measured plant height (PH) (image data), shoot length (SL) (manual data), and fresh weight (FW) of 162 recombinant inbred lines derived from a cross between ‘Milyang23’ and ‘Gihobyeo’ (MGRILs) for analysis of QTLs. All components collected were following normal distribution (data is not shown). For PH was as accurate as SL based the subsampling residuals variance (Table 1). Significant and high correlations were found between the image data and manual data (Table 2). Among the manually collected components, SL and FW showed significant correlation (0.66), which is indirectly consistent with the result using dry weight instead of fresh weight in 16-day old seedlings^4^.

**Table 1:** Results from F test for variances.

df 1	df 2	F	P value
1187	1272	880.1829	1

It tests the null hypothesis that residual variance of plant height and shoot length are equals.

**Table 2:** Correlations among traits measured.

	Plant height	Shoot length	Fresh weight
Plant height	1	0.88 *P* < 0.0001	0.66 *P* < 0.0001
Shoot length		1	0.62 *P* < 0.0001

Pearson’s Correlation among least square means of plant height, shoot length, and fresh weight.

**Figure 1:**
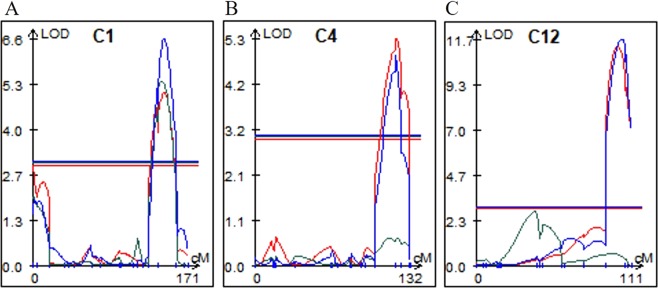
Genetic map showing initial growth related QTLs in the current population. Abbreviation of each trait means as follow. *qPH*, projected plant height in red; *qFW*, fresh weight in blue; *qSL*, shoot length in green. C1, C2, and C12 mean chromosome numbers.

Seven QTLs of the 3 components, PH, SL, and FW, collected in this study for seedling vigor were detected in 2-week old seedlings. Detailed information is in Fig. 1. Among them, 3 QTLs of PH were from image analysis, which was found on chromosome 1, 4, and 12 as 3 QTLs of SL from manual measure were. This is consistent with the previous results using matured plants on chromosome 1^3,5–9^, on chromosome 4^6^, and on chromosome 12^6,10^. Interestingly, QTL of FW was found only on chromosome 1 in the same region as PH and SL.

Notably, mesocotyl length of 10 days old seedlings^11^ and leaf sheath length and culm length of 30 days old seedlings^12^ were also associated with the same region on chromosome 1 for PH; while QTLs of PH of matured plant were detected on chromosome 4 and chromosome 12^6^.

PH in rice is determined by the top leaf length in conventional method. However, it is more complicated trait. For matured plants, PH of can be determined by the length of culm (node and internode) and top leaf attached to culm. Leaf length can be divided by leaf blade and leaf sheath. Culm is comprised of nodes and internodes, which is wrapped by leaf sheath. Thus, culm and leaf sheath length should be highly correlated. Mesocotyl is extended part from root to be connected with culm, which has a stem-like function. However, in shoot establishment stage, seedling above ground is dissected by primary leaf and secondary leaf, and mesocotyl. Thus, PH in young seedling stage is determined by the length of mesocotyl, shooting leaf, and culm. Interestingly, the overlapping peaks of QTL in matured plants and young seedlings associated with PH was located in chromosome 1. In addition, the biomass, FW, shared the same peak of QTL only on chromosome 1, which should be because the determinant factors for plant weight are culm and mesocotyl rather than top leaf. The recent result for PH^3,9^ in matured plants using high throughput phenotyping method showed very strong peak on the same region on chromosome 1. The former utilized the sonic sensor which should be more related with the culm length than with top leaf because it measures the length from the ground to the top not the stretched leaf length. The latter could not detect the leaf length bent over the other side in the three dimension which could be the longest leaf. These cues lead to presume that those peaks of QTL in chromosome 4 and 12 might be associated with the length of leaf blade on the top node which is connected with culm. Therefore, the image analysis appears to be able to differentiate the length of culm and leaf in the very early stage, 2-week old seedlings because the leaf is erect in this stage which is easy to detect in two-dimension.

The peak of QTL on chromosome 1 was known area where *semi-dwarf 1* (*sd1*) gene is located. This gene is associated with one of the most important determinants of plant height, gibberellin (GA)^13^ as confirmed in many other studies as well. Thus, this gene seems to be the major component for PH and it seems to be responsible for especially for culm size due to the reason stated above. The fact that it could be detected in the early stage may imply that this gene is activated from early to matured stage.

This QTL region on chromosome 1 seems to be stable across environments^8^. It is also associated with biomass, FW, which was consistent with previous studies^3,7,8^. Further, it is known to be associated with root traits including maximum root number^6^, root dry weight per tiller and root and shoot ratio^14^, panicle traits such as panicle length, number of panicle per plant, and panicle exertion^6^, and yield traits, for instance, grain yield and harvest index^3,7^, and grain weight^7,10^. It was even associated with several indexes for photosynthesis using remote sensors^3^. Hittalmani *et al*. (2003)^7^ concluded that peaks of QTL for different traits, including projected plant height, panicle number, and panicle length suggest that pleiotropism and or tight linkage of those traits. In that study, traits such as harvest index, number of panicles, panicle length, and 1000 grain weight were very stably detected in the same QTL region on chromosome 1 across different environments as well as plant height. A few year later, Ashikari *et al*. (2005)^5^ suggested that *sd1* allele could have pleiotropic effects on grain number and Tanger *et al*. (2017)^3^ confirmed that PH and yield are in pleiotropic QTL region, which is matched with the result on the chromosome 1. Furthermore, the fact that this QTL region is associated with many important agronomic traits, especially yield, in rice in highly reliable manner across different environments is important for breeding using high potential selection technique area, phenomics, using relatively easy trait to collect, PH.

## Conclusions

So far, the yield related studies have used matured plants due to the technical limit and lack of studies of relationship between traits in early and matured stage. Even after recent emergence of phenome, the application for selections for breeding purpose was not presented. However, current study could detect 3 QTLs associated with PH in only 2 weeks old seedlings, which was consistent with many other reports using various genetic resources in matured stage. This means that the “phenome-assisted selection” can be applied for yield trait in rice in the very early stage. This phenome-assisted selection is crucial advance for breeding purpose in terms of the following aspects other than earliness compared to genomic method. First, it does not destruct any tissue to collect data. Second, it does not need extra cost once the facility and equipment are set up. Third, the data process is very fast. Last but not least, it does not require the full genome sequencing or QTLs studies. Over all, it is much faster and less-cost method with accuracy even compared with marker assisted selection, which could accelerate breeding cycles.

## Methods and Methods

### Plant growth and experimental materials

In this study, 162 recombinant inbred lines derived from a cross between ‘Milyang23’ and ‘Gihobyeo’ (MGRILs) were used for analysis of QTLs related with initial growth rate. These populations were progressed more than F25 generation from F2 of two parents with single seed decent (SSD) methods. Genetic analysis and physical map were made from InDel, STS, and RTM markers^15^. Because tongil type cultivar ‘Milyang23’ and japonica type cultivar ‘Gihobyeo’ showed different germination speed, they were induced in low temperature of 23 °C for 3 days after hot water dipping in 60 °C for 10 minutes. Germinated seeds were grown in 50-hole seedling tray. MGRILs was grown in day length of 14 h light/10 h dark for 2 weeks, and also growth temperature (32 °C in day and 22 °C in night) and humidity (~52%) were constantly maintained. To eliminate edge effect, outermost plants were removed (Fig. S1)

### Survey of growth patterns in MGRILs

Growth patterns of plants were searched with shoot length, fresh weight, and dry weight in 2 weeks day after sowing (DAS). They were actually measured with a ruler and balance for 8 plants per lines. Shoot length was measured from the ground to the longest leaf tip, and the measurement of fresh weight was used to shoot part of above ground. Meanwhile dry weight was weighed after dry of 70 °C for 5 days in drying oven. The plants grown for two weeks on a 50-hole tray were placed on a car equipped with adaptors one by one, and then rotated by a conveyor belt to take photographs sequentially.

### Image acquisition

Image of rice phenotypes were analyzed with matlab program after shooting through 3D scanalyzer imaging system (LemnaTec, Germany). RGB (Red, Green, Blue) images of Plants had the resolution of 6,576 × 4,384. At this time, light condition was constantly set with camera gamma value, 65; gain value, 1000; exposure time, 38,000 *μs*. Each line of MGRILs was photographed in maximum area of plants body (Fig. S1).

### Algorithm application on image analysis

Acquired images were transferred into PNG files, and they were loaded through Matlab program (MathWorks, USA, https://www.mathworks.com/products/matlab.html). First of all, RGB images were transformed into HSI (Hue, Saturation, Intensity) and Lab (L for lightness and a and b for the colour opponents green–red and blue–yellow) channel, and performed background removal. To easily calculate change of colour space, each channel was converted into range of 0 to 255 as follows;

$${\rm{Y}}{\_}_{{{\rm{a}}}^{\ast }}=\{({{{\rm{a}}}^{\ast }}_{{\rm{LAB}}}+100)/200\}\times 255$$

$${\rm{Y}}{\_}_{{{\rm{b}}}^{\ast }}=\{({{{\rm{b}}}^{\ast }}_{{\rm{LAB}}}+100)/200\}\times 255$$

$${\rm{Y}}{{\rm{\_}}}_{{\rm{H}}}=({{\rm{H}}}_{{\rm{H}}{\rm{S}}{\rm{I}}}/360)\times 255$$

- Y__a*_: The value obtained by changing the range of a* channel of LAB color space from 0 to 255
- Y__b*_: The value obtained by changing the range of b* channel of LAB color space from 0 to 255
- Y__H_: The value obtained by changing the range of HUE channel of HSI color space from 0 to 255
- a^*^_LAB_: The a* channel value of the LAB color space
- b^*^_LAB_: The b* channel value of the LAB color space
- H_HSI_: The HUE channel value of the HSI color space

HIS-H, one of separated hue channel, was used to colour range from yellow to green colour region. Lab-a was used to region of green colour. On the other hand, Lab-b was used to region of yellow colour. After that, ROI (region of interest) were extracted through masking from background removed images. Extracted images eliminated noise to median filer^16^, and multi-step morphology was continuously applied in order to clarify ROI structure using erosion and dilation filer^17,18^ Fill area is filtering method that paints a region surrounded by dots in an image. It is used to fill in the interior of a plant image that has been emptied by image conversion after image capture^19,20^. Colour classification and binary images were obtained using the noise and the filtered images. Using the obtained binary image, the number of pixels and the projected plant height were extracted from rice images (Fig. S2, S3).

### QTL analysis and correlation coefficient survey

For the QTL analysis, we used genetic maps of MGRILs that were originally written with 224 PCR-based markers^15^. This was done using the Windows QTL Cartographer V2.5^21^ program. Significant LOD (logarithm of the odds) threshold was adopted by performing 1000 permutations at 95% significance level for each trait. Correlation coefficients between the measured values (plant length, biomass weight, dry weight) and image analysis values (number of pixels, projected plant length) of the growth characteristics of rice were examined.

## Supplementary information

Supplemetary Figures
