# Supplementary material for: The opening of phenome-assisted selection era in the early seedling stage
Source: Sci Rep. 2019 Jul 9;9:9948. doi: 10.1038/s41598-019-46405-3 (PMC6616326; doi:10.1038/s41598-019-46405-3)
Supplement: Supplementary file 1 — Supplemetary Figures [file 41598_2019_46405_MOESM1_ESM.pdf]

The opening of phenome-assisted selection era in the early seedling stage

Song Lim Kim[1†], Yong Suk Chung[2†], Renato Rodrigues Silva[3], Hyeonso Ji[1], Hongseok Lee[1], Inchan Choi[1], Nyunhee Kim[1], Eungyeong Lee[1], JeongHo BAEK[1], Gang-Seob Lee[1], Taek-Ryoun Kwon[1], and Kyung-Hwan Kim[1]*

[1]National Institute of Agricultural Sciences, Rural Development Administration (RDA), Jeonju 54874, Korea

[2]Department of Plant Resources and Environment, Jeju 63243, Jeju National University

[3]Institute of Mathematics and Statistics, Federal University of Goiás, Goiânia, Brazil

*Corresponding author. Email: biopiakim@korea.kr

[†]Equal contributions

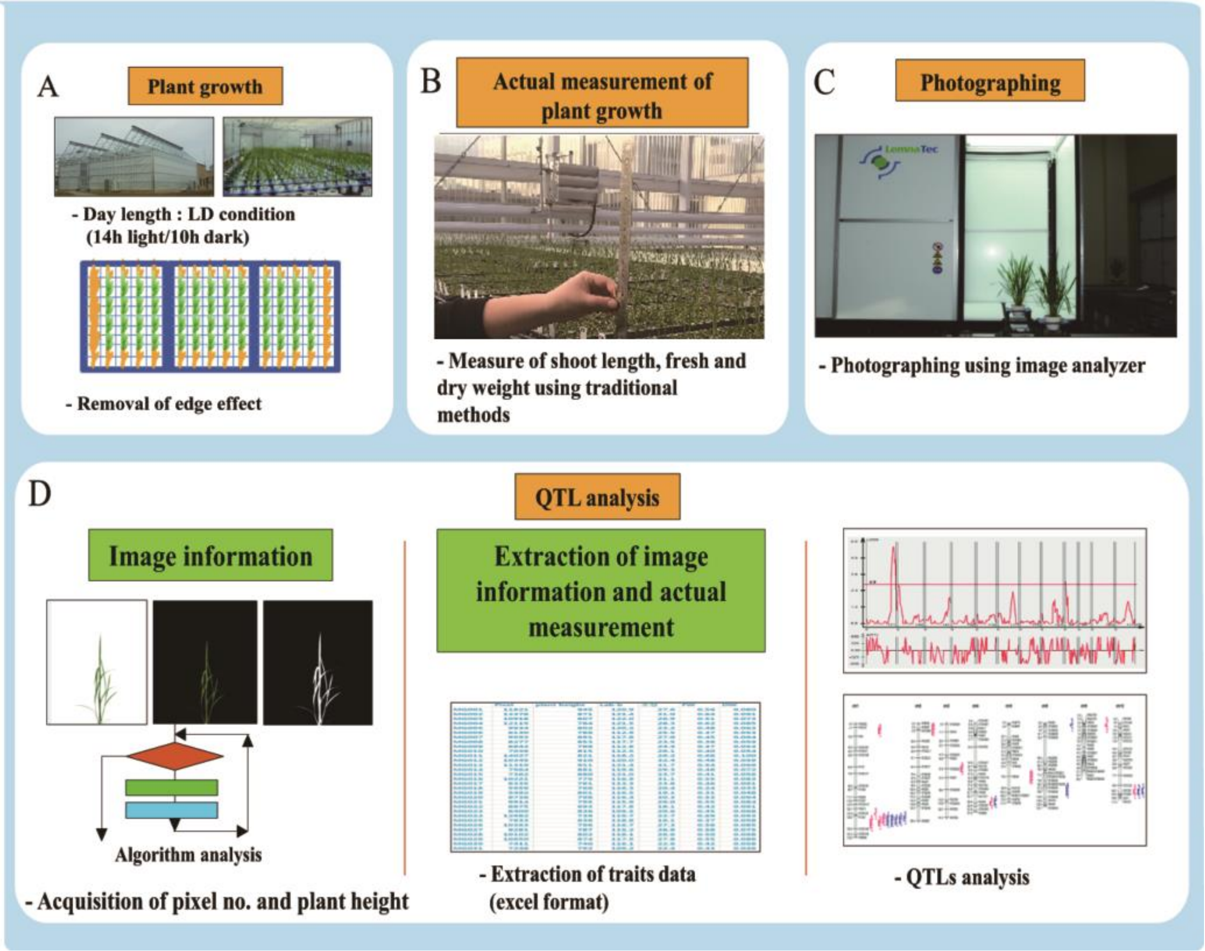

Fig S1. Analysis process of QTLs related initial growth rate using Plant phenomics technology.

(A) Plant growth in phenotype analyzing greenhouse. (B) Actual measurements of MGRILs growth by traditional methods. (C) Photographing using image analyzer. **(D)** QTL analysis through image and growth information.

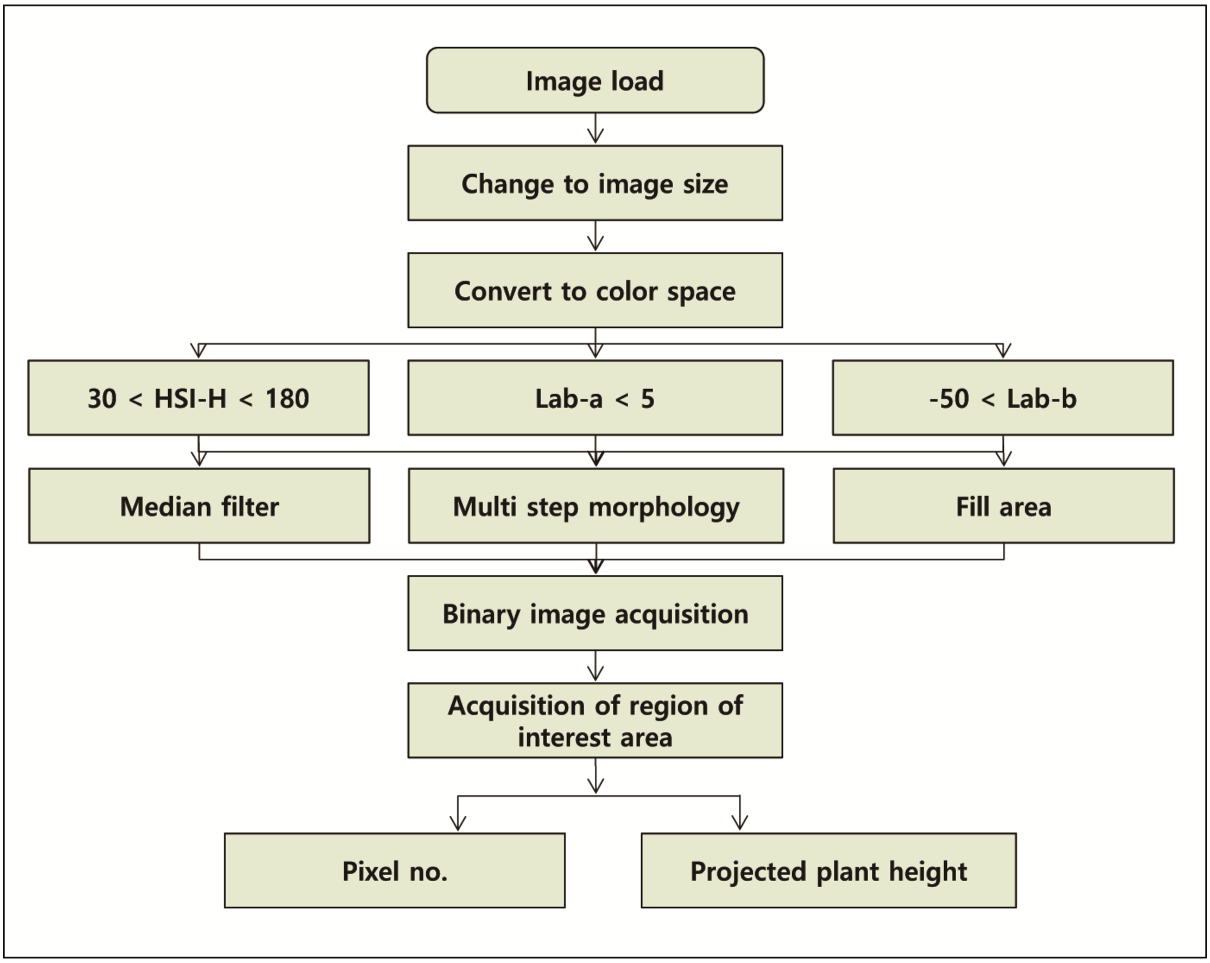

Fig. S2. Algorithm of image analysis.

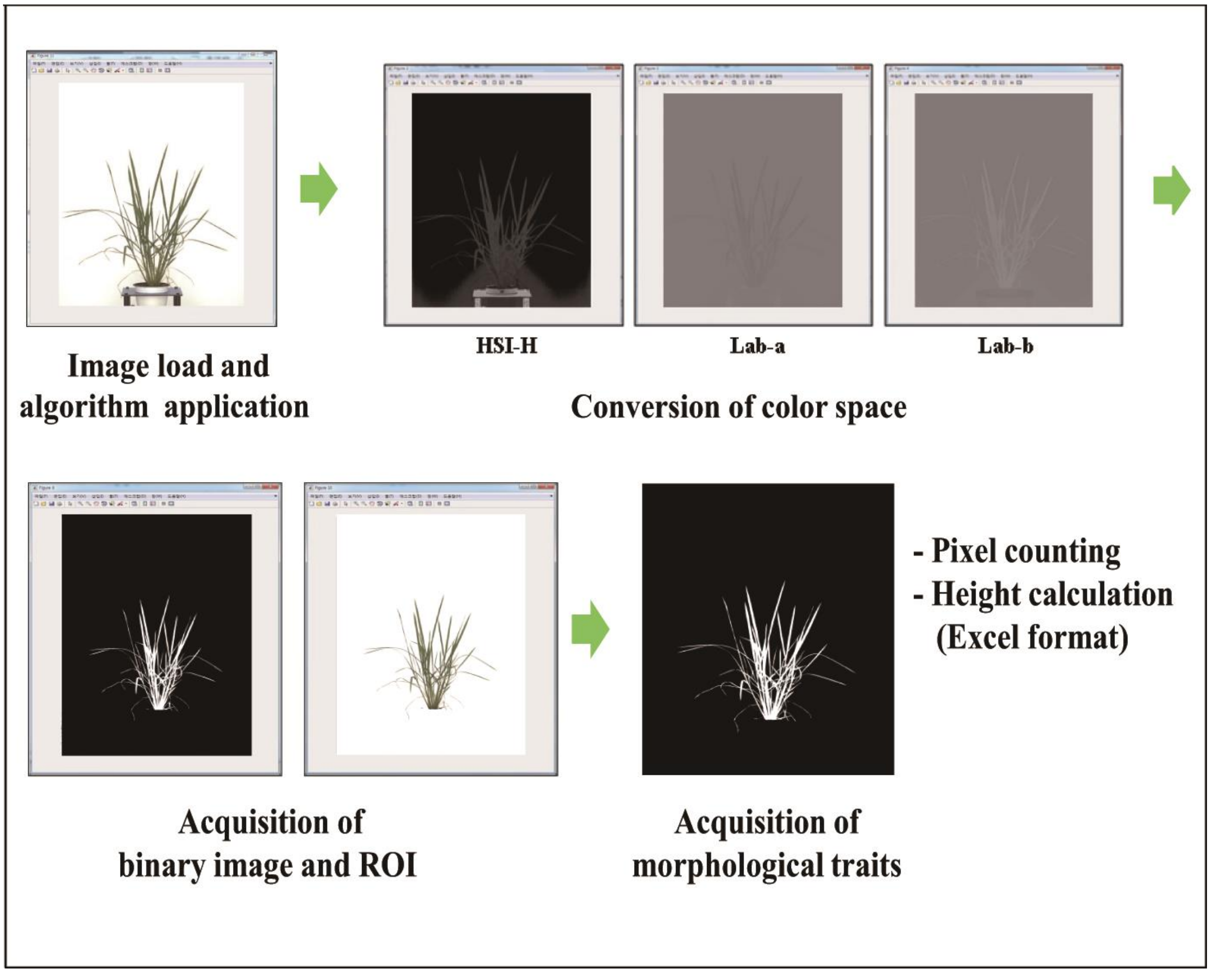

Fig. S3. Detailed acquisition process of image information.
